# Translation and validation of M.D. Anderson Symptom Inventory-Thyroid Cancer module in Chinese thyroid cancer patients: a cross-sectional and methodological study

**DOI:** 10.1186/s12885-022-09995-2

**Published:** 2022-08-26

**Authors:** Zi-yi Hu, Ju-xiang Gou, Ming Cai, Yue-er Zhang

**Affiliations:** 1 Thyroid Surgery Department, West China Hospital, Sichuan University, Chengdu, 610041 Sichuan China; 2 West China School of Nursing, Sichuan University/Thyroid Surgery Department, West China Hospital, Sichuan University, Chengdu, China; 3 Pain Department, West China Hospital, Sichuan University, Chengdu, 610041 Sichuan China

**Keywords:** Thyroid cancer, Symptom, Quality of life, M.D. Anderson Symptom Inventory-Thyroid Cancer module, Reliability, Validity

## Abstract

**Aim:**

To translate and validate the Chinese version of the MDASI-THY among thyroid cancer patients.

**Background:**

The M.D. Anderson Symptom Inventory-Thyroid Cancer module (MDASI-THY) is one of well-validated instruments for thyroid-specific symptom assessment. To date, the instrument has not been used in China.

**Methods:**

After standard forward- and back-translation procedures, two instruments, the Chinese version of MDASI-THY and the European Organization for Research and Treatment of Cancer QLQ C30, were answered by 309 thyroid patients. The content, convergent discriminant validity and reliability of the MDASI-THY were evaluated.

**Results:**

The scale of content validity index (S-CVI) and the item of content validity index (I-CVI) of the instrument were over 0.80. There were significant relationships between MDASI-THY and EORTC QLQ-C30 (r range, 0.139 ~ 0.766, -0.759 ~ -0.461, *p* < 0.001). Symptoms were severer for patients underwent surgical treatment (*Z* = -9.999, *p* < 0.001). The Cronbach’s alpha was 0.966 (between 0.954 and 0.827 for subscales). Most symptom items had moderate to high interitem correlations (r range, 0.297 ~ 0.773).

**Conclusions:**

The Chinese version of MDASI-THY demonstrated favorable validity and reliability. It can be used in development of symptom management program in thyroid cancer patients in China.

**Relevance to clinical practice:**

Healthcare providers can apply this instrument to assess Chinese thyroid cancer patients to increase the understanding of their symptom experience, resulting in a better symptom management.

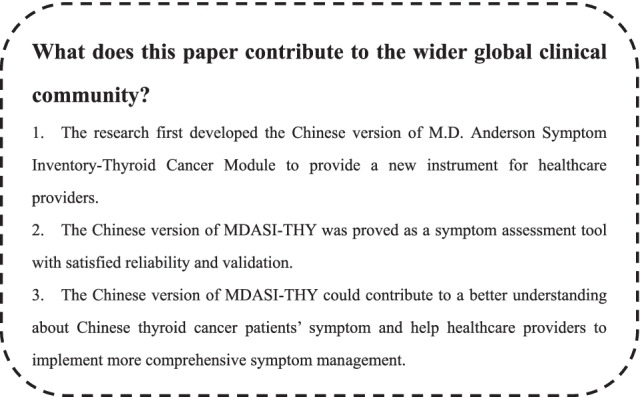



## Introduction

Incidence of thyroid cancer has increased rapidly in China even over the world in recent decades [1]. Primary malignant neoplasms of the thyroid gland can be divided into four categories, namely papillary, follicular, medullary or anaplastic, mainly the papillary [2]. Symptoms of thyroid cancer patients could arise by the disease or by the treatments such as the surgery and ^131^I therapy [3–6]. It is essential and benefit to perform symptom assessment throughout the course of the disease to help healthcare providers to know more about the severity and pattern of a patient’s symptom experience. Thereby, the healthcare providers could make more proper decisions about symptom management and treatment, ideally can better control symptoms [7]. Furthermore, better symptom control can improve thyroid cancer patients’ health-related outcomes.

Prior studies assessed the symptoms of thyroid cancer patients by the general scales like Edmonton Symptom Assessment Scale [8]. However, thyroid cancer patients experience several specific symptoms despite the common symptoms like hoarseness and numbness of hands and feet [5]. We need such a mature instrument to apply in the symptom evaluation of thyroid cancer patients. The M.D. Anderson Symptom Inventory-Thyroid Cancer module (MDASI-THY) is based on the M. D. Anderson Symptom Inventory and completed with the thyroid-specific items [7]. The original version of the MDASI-THY has been validated as a favorable instrument to evaluate the symptom severity and interference of thyroid cancer patients [4, 7]. Regrettably, the instrument has not been developed into Chinese version through cross-cultural adaptation.

The objectives of this study were to translate and validate an instrument for assessing thyroid cancer patients’ symptoms in Chinese setting, to identify the MDASI-THY for reliability and validity to translate it into a local Chinese language. We hypothesized the Chinese version of MDASI-THY would have favorable validity and reliability.

## Method

### Samples and setting

The cross-sectional, methodological study was conducted in a tertiary class-A general hospital from May 2020 to August 2020. Thyroid cancer inpatients in the thyroid surgery department were enrolled to generate a convenience sample.

The inclusion criteria were: (i) age ≥ 18 years; (ii) diagnosed as thyroid cancer by the fine needle aspiration cytology. The exclusion criteria were: (i) not able to communication in Chinese; (ii) unwilling to the study; (iii) had other cancers and distant metastases; (iiii) combined with a history of mental illness.

### Methods and variables

#### Demographic and medical information

Demographic data collected included age, gender, educational background, marital status and employment status. Medical information regarding tumor pathology type and treatment history.

### M.D. Anderson Symptom Inventory-Thyroid Cancer module (MDASI-THY)

The MDASI-THY was developed from the previous general modules of MDASI [7]. The scale was combined with two parts. The first part was a list of 19 items about the symptom severity which include 13 core symptoms as pain, fatigue, nausea, disturbed sleep, emotional distress, shortness of breath, difficulty remembering, lack of appetite, drowsiness, dry mouth, sadness, vomiting and numbness or tingling, 6 thyroid-specific symptoms like hoarseness, problem with feeling hot, problem with racing heartbeat, problem with feeling cold, difficulty swallowing and diarrhea or loose stools. 6 symptom-related interference items (general activity, mood, normal work (including both work outside the home and housework), relations with other people, walking ability and enjoyment of life) compose the second part which are consistent with other modules and they describe how much the symptoms have interfered with different aspects of the patient’s life during the past 24 h. According to the study of Gning [7], the interference subscale can be subdivided into 2 component scores: WAW (mean of the physical items, i.e., walking ability, activity and work) and REM (mean of the affective items, i.e., relations with others, enjoyment of life and mood). Each symptom and interference item are rated on an 11-point (0–10) scale, with 0 being ‘not present’ and 10 being ‘as bad as you can imagine’.

### European Organization for Research and Treatment of Cancer Quality of Life Questionnaire-Core 30 (EORTC-QLQ C30)

EORTC QLQ-C30 is the core scale of evaluating the quality of life and widely used worldwide for cancer patients [9–12]. EORTC QLQ-C30 comprises 30 items and each raw score (RS) is converted into standard score (SS) of 0–100 by linear formula, with higher score indicating better performance. The instrument contains five functional sub-dimensions (physical, role, emotional, cognitive, and social functioning), an overall health sub-dimension, three symptom sub-dimensions (fatigue, nausea and vomiting, pain), six individual items related to symptoms that are frequently occurred in cancer patients (diarrhea, constipation, insomnia, poor appetite, dyspnea, and financial difficulties). The five functional sub-dimensions were calculated by the formula of SS = [1-(RS-1)/R]*100, and the rest sub-dimensions were converted by the formula of SS = [(RS-1)/R]*100. R presented the full range of the item score. The higher the SS, the better the functions and global quality of life (QoL), otherwise the more severe the symptom subscales and individual items.

### Translation process

The procedures of forward- and back-translate the Chinese version of MDASI-THY followed the translation and cultural adaptation guidelines prescribed in the previous study [13, 14] and performed standard validity and reliability assessment [15].

At the beginning, we emailed to M.D. Anderson Cancer Center (MDACC) to obtain the authorization. We sent the reports of transitions steps to MDACC to make sure there was no major change during translation process. Followed by the permission, two researchers who had different professional backgrounds completed the first step dependently (forward-translation procedures) to translate the MDASI-THY from English into Chinese. After that, the two researchers compared the inconsistencies of two translated versions of the MDASI-THY regarding ambiguities and discrepancies of words, sentences and meanings [14]. The step could only end after two researchers made the consensus. Finally, a researcher who was unknown the original version of the MDASI-THY accomplished the back-translation of the first Chinese version. At last, comparison among the original version, the back-translated version and the first Chinese version for conceptual consistency by the above three translators until no ambiguities or discrepancies were found.

**Fig. 1:**
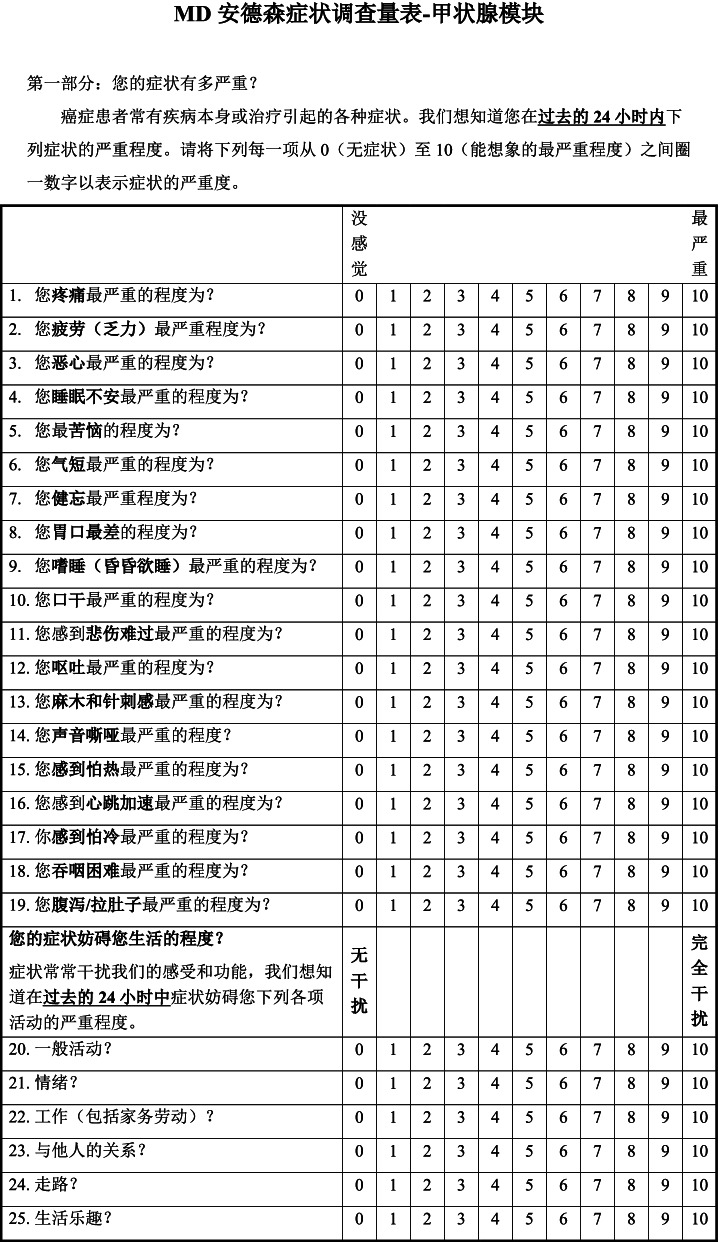
The original version and Chinese version of the MD Anderson Symptom Inventory-Thyroid Cancer Module (MDASI-THY)

The Chinese version of MDASI-THY was cognitive debriefed to evaluate the instructions expression and the items’ understanding of the instrument for clarity. Twelve thyroid cancer patients who were informed consent and had different socio-demographic and clinical characteristics were investigated (66.7% female, 58.3% married, 83.3% underwent surgical treatment, 100% Papillary carcinoma of thyroid). They were investigated to answer the Chinese version of the MDASI-THY according to their own symptom experience. After above, participants were invited to rate the instructions expression and the items’ understanding as clear or unclear. If participants rated the instruction or item as unclear, they would be interviewed about the suggestions about the promotion. The revision must be done until the rate of unclear items was found under 20%. So far, the final Chinese version of MDASI-THY was established (Fig. 1).

### Declaration

Standard informed consent process was conducted. The study was approved by the Institutional Review Board of West China Hospital (IRB #2021[362]). Each participant had a face-to-face meeting with the researchers during which the researchers ensured that the patients met the study criteria and explained the study in detail. The researchers provided enough time for the patients to ask questions. Each participant signed the written informed to the study. Protection of human subjects was ensured by following the guidelines set forth by the Institutional Review Board.

### Psychometric evaluation

#### Reliability

Internal consistency was evaluated using Cronbach’s alpha and the correlation of each item in the MDASI-THY with the total score. Cronbach alpha values over 0.70 indicated a satisfied internal consistency reliability [15].

#### Validity

Content validity, convergent validity, and discriminant validity were used to evaluate the validity of the Chinese version of MDASI-THY. The content validity was assessed by six experts who all had medical or nursing master's degree or above, and had at least 10 years work experience in healthcare of the thyroid cancer patients. The experts assessed the items by giving scores ranging from 1 to 4, which represented “highly relevant”, “relevant”, “irrelevant” and “highly irrelevant”, respectively. Content validity index at the item level (I-CVI) was calculated by the number of the experts who rate 1 or 2 divided by the total number of experts. Content validity index at the scale level (S-CVI/UA) equals the number of the relevant items divided by the total number of the scale items. S-CVI/Ave was the mean value of all items’ I-CVI of the MDASI-THY. S-CVI/UA over 0.8, or S-CVI/Ave over 0.9 indicate the scale has a good content validity [16]. Convergent validity was calculated by analyzing the relationships between the MDASI-THY score with the subscales/items of EORTC QLQ-C30 scores. Discriminant validity could show the ability of the scale to distinguish the different groups of thyroid cancer patients.

### Statistical analysis

PASW (Predictive Analytics Software, IBM) statistical 18.0 software was applied for data analysis. Descriptive statistics analysis was performed in demographic and clinical variables. Means, standard deviation (SD) and quantiles [M(Q1, Q3)] were used to describe the continuous variables according to whether the variables followed the normal distribution or non-normal distribution, respectively. Frequency and percentage were analyzed to describe the categorical variables. The correlation between MDASI-THY and EORTC QLQ-C30 was carried out by Spearman’s correlation coefficients as a result of the non-normal distribution of the data. Mann–Whitney U test of nonparametric test was used to compare MDASI-THY scores between different groups of thyroid cancer patients. The internal consistency reliability was tested by calculating the Cronbach’s alpha. To measure the interitem correlation, Spearman correlation analysis was performed among the items of MDASI-THY. *p* ≤ 0.05 was considered statistically significant.

## Results

### Participant characteristics

**Fig. 2:**
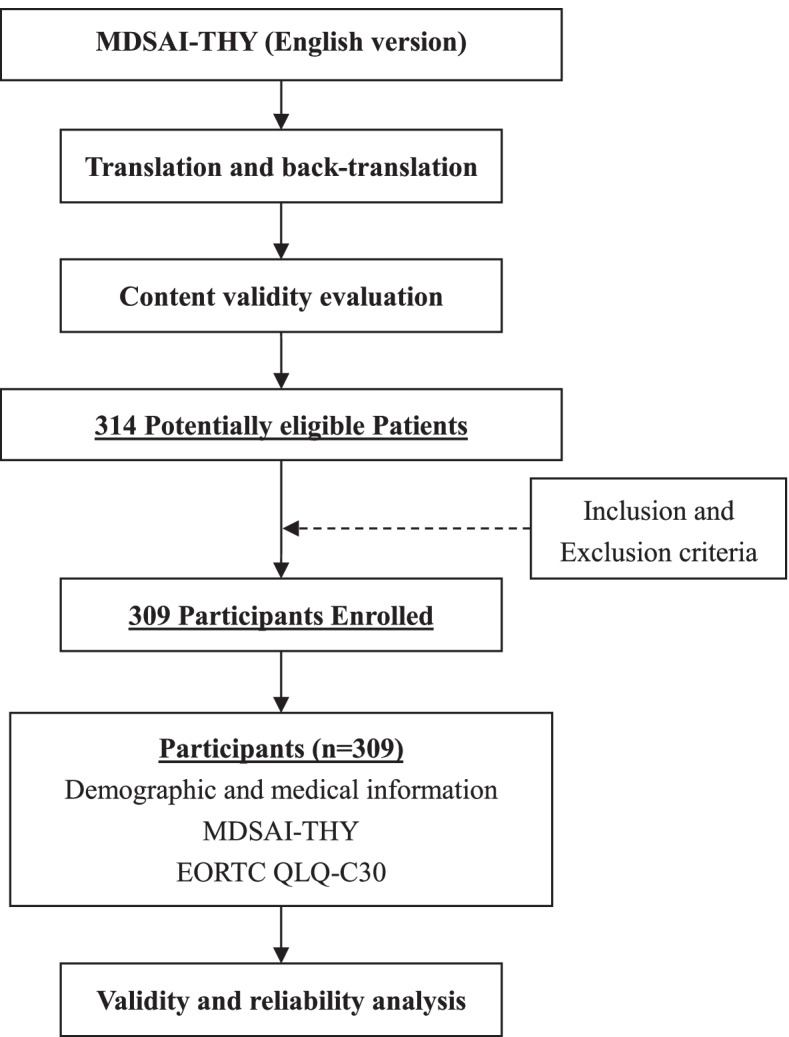
Flow diagram

A total of 309 thyroid cancer patients joined our research and completed the investigation (Fig. 2). All questionnaires were complete. The age of the participants was 37.02 ± 8.71 years and ranged from 18 to 56. For gender composition, 73.8% of the participants were female. The majority was married (79.6%), had higher than a high school education (74.8%) and was employed (79.6%). Besides, most (99.0%) participants had papillary thyroid carcinoma, and 3 had medullary thyroid carcinoma (1.0%). There were 244 patients (79.0%) who received surgery and 65(21.0%) who haven’t received treatment.

### Cognitive debriefing

For the pilot study, twelve participants agreed the Chinese version of MDASI-THY was easy to understand, and not difficult to fill in. All symptom items were close to their own feelings and it was effortless to recall the symptom experience.

### Validity of the MDASI-THY

#### Content validity

After six experts evaluating, we combined the scores value into two categories, namely relevant and irrelevant. 21 of 25 items got I-CVI of 1.00, and the rest of 4 items (Item 7, 19, 22 & 24) got 0.83. All I-CVI values were over 0.80, showing the scale had a satisfied content relevance. The S-CVI/UA of the MDASI-THY was calculated as 0.84 and the S-CVI/Ave was 0.97, which met with standard of previous study, and indicating a content validity.

#### Convergent validity

The Spearman correlation coefficients between the subscales of MDASI-THY and the subscales of EORTC QLQ-C30 were shown in the Table 1. The symptom severity items, core symptoms, thyroid cancer symptoms, interference items, WAW and REM were negatively correlated with global QoL and functional subscales. The symptom severity items, core symptoms, thyroid cancer symptoms, interference items, WAW and REM were positively correlated with the symptom subscales and individual items except for the correlation coefficient between diarrhea and REM.

**Table 1:** The convergent validity of the MDASI-THY score (*n* = 309)

EORTC QLQ-C30 subscales	Symptom severity items		Core symptoms		Thyroid cancer symptoms		Interference items		WAW		REM	
	*r*	*p*	*r*	*p*	*r*	*p*	*r*	*p*	*r*	*p*	*r*	*p*
Global QoL	-0.550	< 0.001	-0.557	< 0.001	-0.496	< 0.001	-0.521	< 0.001	-0.512	< 0.001	-0.478	< 0.001
Physical functioning	-0.759	< 0.001	-0.751	< 0.001	-0.733	< 0.001	-0.654	< 0.001	-0.659	< 0.001	-0.584	< 0.001
Role functioning	-0.590	< 0.001	-0.586	< 0.001	-0.574	< 0.001	-0.600	< 0.001	-0.591	< 0.001	-0.559	< 0.001
Emotional functioning	-0.509	< 0.001	-0.517	< 0.001	-0.466	< 0.001	-0.555	< 0.001	-0.461	< 0.001	-0.581	< 0.001
Cognitive functioning	-0.709	< 0.001	-0.711	< 0.001	-0.669	< 0.001	-0.627	< 0.001	-0.601	< 0.001	-0.618	< 0.001
Social functioning	-0.644	< 0.001	-0.645	< 0.001	-0.631	< 0.001	-0.661	< 0.001	-0.635	< 0.001	-0.599	< 0.001
Fatigue	0.765	< 0.001	0.766	< 0.001	0.726	< 0.001	0.660	< 0.001	0.658	< 0.001	0.585	< 0.001
Nausea and vomiting	0.395	< 0.001	0.391	< 0.001	0.377	< 0.001	0.362	< 0.001	0.359	< 0.001	0.349	< 0.001
Pain	0.624	< 0.001	0.619	< 0.001	0.603	< 0.001	0.573	< 0.001	0.582	< 0.001	0.521	< 0.001
Dyspnea	0.526	< 0.001	0.519	< 0.001	0.501	< 0.001	0.478	< 0.001	0.488	< 0.001	0.458	< 0.001
Insomnia	0.636	< 0.001	0.637	< 0.001	0.591	< 0.001	0.577	< 0.001	0.535	< 0.001	0.530	< 0.001
Appetite loss	0.537	< 0.001	0.537	< 0.001	0.514	< 0.001	0.493	< 0.001	0.490	< 0.001	0.464	< 0.001
Constipation	0.413	< 0.001	0.409	< 0.001	0.407	< 0.001	0.380	< 0.001	0.369	< 0.001	0.360	< 0.001
Diarrhea	0.207	< 0.001	0.199	< 0.001	0.207	< 0.001	0.116	0.042	0.139	0.014	0.043	0.447
Financial difficulties	0.476	< 0.001	0.484	< 0.001	0.448	< 0.001	0.473	< 0.001	0.485	< 0.001	0.456	< 0.001

#### Discriminant validity

As results were shown in Table 2, the thyroid cancer patients who underwent surgical treatment had more severe symptom and symptom interference than who had no surgery. It declared that MDASI-THY had a good discriminant validity that could distinguish the of different level of symptom experience in thyroid cancer patients with different characteristics groups.

**Table 2:** The discriminant validity of the MDASI-THY score (*n* = 309)

Groups	Symptom severity of MDASI-THY			Symptom interference of MDASI-THY		
	M(Q_1_,Q_3_)	*Z*	*p*	M(Q_1_,Q_3_)	*Z*	*p*
Surgery		-9.999	< 0.001		-8.497	< 0.001
No (*n *= 65)	0(0,0)			0(0,0)		
Yes (*n* = 244)	1.47(0.38,2.62)			0.67(0,2.33)		

### Reliability of the MDASI-THY

The Cronbach's alpha coefficient of MDASI-THY was 0.966, and the Cronbach's alpha coefficients of all the subscales were range from 0.827 to 0.954, suggesting the scale has a good internal consistency (Table 3). Interitem correlation of the MDASI-THY was shown in Table 4, and all 19 symptom items showed a strong relationship. Most correlations between two symptoms indicated a moderate to high value.

**Table 3:** The reliability of the MDASI-THY score (*n* = 309)

MDASI-THY subscales	Cronbach’s alpha	Items, n
Symptom severity items	0.954	19
Core symptoms	0.943	13
Thyroid cancer symptoms	0.827	6
Interference items	0.929	6
WAW	0.883	3
REM	0.872	3

**Table 4:** Interitem correlation for the MDASI-THY symptoms (*n* = 309)

	pain	fatigue	nausea	disturbed sleep	distressed	Short breath	remember	appetite	frowsy	dry mouth	sad	vomiting	numberness	hoarseness	feeling hot	racing heartbeat	feeling cold	swallowing
fatigue	0.773^**^																	
nausea	0.554^**^	0.541^**^																
disturbed sleep	0.653^**^	0.714^**^	0.484^**^															
distressed	0.649^**^	0.685^**^	0.539^**^	0.602^**^														
short breath	0.501^**^	0.629^**^	0.586^**^	0.525^**^	0.594^**^													
remembering	0.521^**^	0.654^**^	0.440^**^	0.554^**^	0.581^**^	0.675^**^												
lack of appetite	0.707^**^	0.712^**^	0.628^**^	0.608^**^	0.661^**^	0.644^**^	0.596^**^											
feeling frowsy	0.707^**^	0.746^**^	0.584^**^	0.623^**^	0.645^**^	0.579^**^	0.584^**^	0.689^**^										
dry mouth	0.743^**^	0.739^**^	0.539^**^	0.589^**^	0.592^**^	0.551^**^	0.593^**^	0.677^**^	0.713^**^									
sad	0.582^**^	0.648^**^	0.599^**^	0.588^**^	0.722^**^	0.661^**^	0.658^**^	0.678^**^	0.630^**^	0.571^**^								
vomiting	0.499^**^	0.487^**^	0.716^**^	0.441^**^	0.498^**^	0.549^**^	0.413^**^	0.599^**^	0.531^**^	0.431^**^	0.603^**^							
numberness	0.646^**^	0.641^**^	0.455^**^	0.568^**^	0.658^**^	0.526^**^	0.568^**^	0.620^**^	0.632^**^	0.621^**^	0.624^**^	0.491^**^						
hoarseness	0.712^**^	0.716^**^	0.511^**^	0.560^**^	0.662^**^	0.606^**^	0.562^**^	0.626^**^	0.663^**^	0.695^**^	0.571^**^	0.469^**^	0.678^**^					
feeling hot	0.528^**^	0.653^**^	0.485^**^	0.558^**^	0.533^**^	0.583^**^	0.615^**^	0.599^**^	0.548^**^	0.589^**^	0.622^**^	0.519^**^	0.540^**^	0.592^**^				
racing heartbeat	0.546^**^	0.638^**^	0.559^**^	0.526^**^	0.583^**^	0.732^**^	0.646^**^	0.611^**^	0.601^**^	0.594^**^	0.682^**^	0.547^**^	0.608^**^	0.566^**^	0.681^**^			
feeling cold	0.557^**^	0.609^**^	0.527^**^	0.530^**^	0.583^**^	0.565^**^	0.541^**^	0.607^**^	0.591^**^	0.520^**^	0.689^**^	0.535^**^	0.503^**^	0.459^**^	0.575^**^	0.650^**^		
swallowing	0.745^**^	0.698^**^	0.529^**^	0.596^**^	0.588^**^	0.515^**^	0.540^**^	0.673^**^	0.675^**^	0.748^**^	0.558^**^	0.439^**^	0.661^**^	0.719^**^	0.545^**^	0.558^**^	0.480^**^	
diarrhea	0.350^**^	0.366^**^	0.334^**^	0.353^**^	0.327^**^	0.389^**^	0.382^**^	0.437^**^	0.471^**^	0.381^**^	0.460^**^	0.422^**^	0.378^**^	0.297^**^	0.422^**^	0.439^**^	0.466^**^	0.330^**^

^*^p < 0.05, ^**^p < 0.01, significant correlation (2-tailed)

## Discussion

In the study, our findings showed that the Chinese version of MDASI-THY had a satisfied validity and reliability among Chinese thyroid cancer survivors, indicating the Chinese version of MDASI-THY is a good instrument to apply in the clinical assessment.

Some scholars have posited that an accepted instrument with sufficient content validity should have a CVI over 0.80 [16, 17]. The I-CVI for all items of MDASI-THY were over 0.80, the S-CVI/UA was 0.84 and the S-CVI/Ave was 0.97, which indicating the instrument were highly relevant with the target measurement content. And the Chinese version of MDASI-THY was judged valid and unambiguous as a tool to apply in symptom assessment of thyroid cancer patients.

As numerous prior researches pointed that severe symptoms were linked to impaired cancer patients’ QoL [12, 18, 19], we calculated the convergent validity by the analysis of the correlations between MDASI-THY and the EORTC QLQ-C30. The correlation coefficients between the MDASI-THY and the EORTC QLQ-C30 were significantly correlative and satisfied, and most subscales were significantly correlated. Because the prevalence of diarrhea was relatively low, diarrhea had a non-significantly correlation coefficient with REM. Therefore, the absolute values of the correlation coefficients between the two instruments ranged from 0.116 to 0.765 (the highest for the fatigue subscale with symptom severity item). According to the results, the Chinese version of MDASI-THY had a good convergent validity and was highly worthy applying in the clinical settings to evaluate thyroid cancer patients’ symptom.

Discriminant validity was verified by the Mann–Whitney U test conducted between two groups of thyroid cancer patients based on disease status (without surgery vs. underwent surgical treatment). Surgical treatment might be an influencing factor of the symptom experience in thyroid cancer patients. In Table 2, patients with surgical treatment reported higher symptom severity and interference scores, because the post-surgical symptoms might be more severe than the pre-surgical symptoms as a result of the surgical wounds or the decreased blood calcium [4, 5, 8]. The MDASI-THY had an optimistic discriminant validity, as it had an excellent ability to discern the thyroid cancer patients underwent/without surgical treatment, which indicating the Chinese version of MDASI-THY had a hopeful ability to distinguish different groups of thyroid cancer patients, which building a foundation for researchers to use the instrument to assess thyroid cancer patients with different clinical characteristics.

The Cronbach’s alphas were 0.827–0.954 (Table 3), which met the acceptable coefficient level of 0.70 [15]. The MDASI-THY demonstrated its satisfactory and sufficient reliability, as indicated by the excellent internal consistency. The Spearman correlation coefficients between the symptom severity and interference items ranged from 0.327 to 0.773, revealing the instrument had good stability and the result cross-validated the previous study [7].

From the above results, the MDASI-THY’s cross-cultural translation and validation from English to Chinese led to a favorable Chinese version that could provide a more specific and valid instrument for thyroid cancer patients’ symptom assessment, bringing more knowledge to the symptom management, and contributing to a better health related outcomes of thyroid cancer patients.

### Strengths and limitations of the study

The Chinese version of MDASI-THY was translated and validated as a favorable instrument to apply in clinical symptom evaluation for thyroid cancer patients. Increased knowledge about symptoms of thyroid cancer patients could lead to better understanding to symptom management, facilitating a better health-related outcome. There were some limitations in the study as we enrolled a sample of thyroid cancer patients from one hospital in the southwest of China, which urged us to validate the findings through applying the instrument in thyroid cancer patients from other provinces in China.

## Conclusion

This study showed the standardized translation process and validated some psychometric properties of the Chinese version of MDASI-THY. The instrument was considered easy to complete and understand. Besides, it was verified that the Chinese version of MDASI-THY had satisfactory content validity, convergence validity and discriminative validity, as well as a good internal consistency. All the evidence we found indicated that the instrument was a reliable and suitable tool for symptom assessment of Chinese patients with thyroid cancer. The Chinese version of MDASI-THY will help healthcare providers to better assess patients’ symptoms, identify changes in patients’ symptoms, and provide more support resource for Chinese thyroid cancer symptom management.

### Relevance to clinical practice

After standard translation and validation of Chinese version of MDASI-THY, a satisfied instrument was developed for nurses. Nurse can apply this instrument to assess Chinese thyroid cancer patients to increase the understanding of their symptom experience, resulting in a better symptom management.

## Data Availability

The data described in this article can be freely and openly accessed at Harvard Dataverse: https://doi.org/10.7910/DVN/0UXEEO.
